# Admission to Be Only by Payment

**Published:** 1888-03-17

**Authors:** 


### ADMISSION TO BE ONLY BY PAYMENT.

THE proprietors of the above home, before commencing the work of another season, beg to inform those through whose recommendation patients have been admitted, and others who may in future apply for tickets, that they have decided that the time has come to make the charge of £1 for each ticket of admission, entitling a patient to stay three weeks in the home. On an application being received by Mrs. Henry Pease, Pierremont, Darlington, a doctor's certificate will be sent, as before, to be filled up and signed; but when it is returned it must be accompanied by a cheque or postal order for £1, before the ticket of admission can be sent. The proprietors earnestly hope that this charge, which represents about two-thirds of the cost of each patient, will not prevent the necessitous and deserving poor, for whom the home was intended, from gaining the benefit, but that, on the contrary, it will be kept full by the kind co-operation of those who estimate the blessing it has been in the past to the sick and suffering.

